# MTA-Based Cements: Biocompatibility and Effects on the Gene Expression of Collagen Type 1 and TGF-*β*1

**DOI:** 10.1155/2022/2204698

**Published:** 2022-03-31

**Authors:** Diana María Escobar-García, María Guadalupe Medina-Rosas, Ana María González-Amaro, Verónica Méndez-González, Héctor Flores, Amaury Pozos-Guillén

**Affiliations:** ^1^Basic Sciences Laboratory, Faculty of Dentistry, San Luis Potosi University, Dr. Manuel Nava Avenue # 2, Zona Universitaria, San Luis Potosí, SLP, 78290, Mexico; ^2^Endodontics Postgraduate Program, Faculty of Dentistry, San Luis Potosi University, Dr. Manuel Nava Avenue # 2, Zona Universitaria, San Luis Potosí, SLP, 78290, Mexico

## Abstract

**Objective:**

This study sought to evaluate the biocompatibility of Neomineral Trioxide Aggregate (Neo-MTA), MTA Repair High Plasticity (MTA-HP), and Mineral Trioxide Aggregate-Angelus white (MTA-Ang) in fibroblasts of human dental pulp.

**Materials and Methods:**

Morphology was evaluated after 24 h of incubation. *LIVE/DEAD* assay and cell adhesion tests were performed at 24 h of treatment. Cell proliferation assays (MTSs) and Annexin V were performed at 48 h incubation with different treatments. The expression of Col-1 and TGF-*β*1 was tested by endpoint PCR at 5 days of treatment.

**Results:**

Morphological changes were observed in all groups. Neo-MTA and MTA-Ang were associated with increased cell viability, and all materials induced apoptosis, with a higher percentage in the MTA-HP group than in the other groups. In the LIVE/DEAD assay, there was more damage to the cell membrane in the group of cells treated with MTA-HP than in the other groups.

**Conclusion:**

Neo-MTA and MTA-Ang presented similar biocompatibility, and both showed greater biocompatibility than MTA-HP. MTA-HP and MTA-Ang increased Col-1A gene expression, and Neo-MTA and MTA-Ang increased TGF-*β*1 gene expression in a similar way.

## 1. Introduction

In cases of dentin lesions resulting from deep cavities, trauma, or iatrogenic origin, a response is provoked that requires the activation and proliferation of pulp cells, and this response is mediated by different signaling molecules involved in the induction of tissue repair [1]. Vital pulp therapy refers to the application of a biomaterial to exposed pulp tissue; the biomaterial must be biocompatible and bioactive and create an environment conducive to tertiary dentin formation [2, 3]. The efficacy of mineral aggregate trioxide (MTA) has been demonstrated during vital pulp therapy; however, research continues in the search for the optimization of its physical/mechanical properties and to improve the biological response [4].

In 2001, the Mineral Trioxide Aggregate-Angelus white (MTA-Ang) was introduced into the market. This traditional formulation has some disadvantages, such as handling difficulty, granular consistency, and long setting time; in addition, the use of bismuth oxide as a radiopacifying agent may promote tooth discoloration [5]. Mineral Trioxide Aggregate Repair High Plasticity (MTA-HP) contains a liquid phase that is formed by water and a plasticizing polymer to mix the powder, which gives it greater plasticity; in addition, replacing bismuth oxide with calcium tungstate prevents tooth discoloration [6]. Neo-MTA contains a gel that facilitates handling and application after being mixed with the powder. The formulation does not pigment and is of quick application [7]. The cytotoxicity of the material used may influence the viability of pulp cells [8], potentially causing cell death. Fibroblasts represent the most abundant cell population within the pulp and perform important functions in regulating the biology and function of dental pulp, both under normal and pathological conditions. Its main action is the secretion of collagen; however, these cells also synthesize growth factors that are involved in the restoration of vascularization, innervation, and regeneration of pulp-dentin complex [1]. Now, dental materials undergo changes that would not only improve the appearance of teeth but also help the regeneration of an injured pulp. However, these improvements may include chemicals that can affect the recovery of dental pulp or adjacent tissues.

This study is aimed at evaluating the biocompatibility and expression of collagen type 1 and TGF-*β*1 of Neo-MTA, MTA Repair, HP, and MTA-Angelus white in human dental pulp fibroblasts.

## 2. Materials and Methods

Fibroblasts of dental pulp (hFDP) were obtained from newly extracted human third molars for orthodontic reasons by the explant growth method, with the approval of the ethics committee of the Faculty of Dentistry (CEI-FE-021-018). Cells were grown in 25 mL culture boxes with 3 mL of culture medium (Dulbecco's Modified Eagle's Medium (DMEM) Sigma-Aldrich, St. Louis, MO, USA) as reported by Escobar-Garcia et al. [8]. Cell cultures with at least 80% confluence were used, which ensures an optimal metabolic state of the cell. The experimental assays were performed in triplicate.

The following cements were used for the study: (i) MTA Repair HP (Angelus, Londrina, PR, Brazil) (MTA-HP), (ii) Neo-MTA (NuSmile, Houston, USA), and (iii) MTA-Angelus white (Angelus, Londrina, PR, Brazil). Under sterile conditions, the materials were mixed according to the manufacturer's instructions and left to stand for 24 h. They were mixed with culture medium supplemented at a concentration of 2.5  mg/mL according to ISO 10993-12-2009 [9] and placed in a roller mixer (SRT6D Stuart) at 60 rpm for 24 h; the eludes obtained were centrifuged at 3000 rpm for 5 min; supernatants obtained were filtered with 0.22 *μ*m membranes and stored in refrigeration until use.

After an incubation period of 24 h, to allow the cells to be in optimal conditions, the culture medium was replaced by the elutes of the materials specified above or with a fresh culture medium for the control group. The morphological characteristics were analyzed by inverted optical microscopy (Leica Microsystems, Wetzlar, Germany) after 24 h of treatment according to ISO 10993 [9] at a magnification of 40x.

Cell proliferation was evaluated by the phenazine methosulfate (PMS) and 3-(4,5-dimethylosazol-2-yl)-5-(3-carboxymethoxyphenyl)-2-(4-sulfophenyl)-2H-tetrazolium (MTS) solution (CellTiter 96 AQueous Non-Radioactive Cell Proliferation Assay, Promega). The cells were cultured at a density of 1 × 10^4^ cells per well in a volume of 100 *μ*L culture medium in 96-well plates and incubated at 37°C under 5% CO_2_ and 95% humidity. After 24 h, the culture medium was removed and replaced with 100 *μ*L of culture medium containing the elutes from the different cements to be evaluated or with a medium without cement (for the negative control group). After 48 h of incubation with different treatments and controls, the test was carried out according to the manufacturer's instructions; then, the cells were incubated at 37°C, 5% CO_2_ and 95% humidity for a period of 4 h. During this period, metabolically active cells bioreduce the salt of MTS to formazan. The test results were read in a microplate reader (Thermo Scientific FC Multiskan, Vantaa, Finland) at 490 nm. The absorbance values of the different treatments evaluated are directly proportional to the number of living cells in culture. All cells treated with the different test cements were compared with negative control cells; these cells are not treated with any sealant, in some tests, there is a positive control (cell death) to which a 30% solution of H_2_O_2_ was added as a cytotoxic agent, and all cements were tested in quintuplicate.

LIVE/DEAD assay was performed with the LIVE/DEAD Viability/Cytotoxicity kit for mammalian cells (AM/ethidium homodimer-1, calcein; Invitrogen, San Francisco, CA, USA), as reported by Escobar-Garcia et al. [8]. The working solution was prepared according to the manufacturer's instructions; the culture medium was then removed, washed 3 times with PBS, added 100 *μ*L of the working solution, and then incubated for a period of 45 min under conditions of darkness at 37°C, 95% humidity and 5% CO_2_. At the end of this time, cell cultures were washed with PBS, and dyed cells were observed by confocal laser scanning microscopy (CLSM) (model DMI4000B; Leica Microsystems, Wetzlar, Germany) to 40x. The cells in contact with the cement to be evaluated were compared with a negative and positive control. The tests were performed in triplicate.

Annexin V assay was performed by flow cytometry using the Annexin V and propidium iodide (PI) (Alexa Fluor 488 Annexin V/Dead Cell Apoptosis Kit, Invitrogen, Carlsbad, CA, USA). The culture media with eluates of the materials were kept in contact with the cells for 48 h. After that time, the monolayers were detached by placing 100,000 cells in 100 *μ*L of 1X Annexin binding buffer, and the assay was continued according to the manufacturer's instructions. The cells were incubated at room temperature for 15 min. The stained cells were analyzed by a FACSCANTO II flow cytometer (Becton-Dickinson, Franklin Lakes, USA) by measuring the fluorescence emission at 530 nm and 575 nm using 488 nm excitation. A total of 5,000 counts were made for each measurement, in triplicate.

A total of 10,000 cells were seeded directly into circular coverslips, which were deposited in culture boxes in 24 wells. Culture medium was added, and the cells were incubated for 24 h at 37°C with 5% CO_2_ and 95% humidity. The culture medium was exchanged for the different eluates, and the cells were incubated. After 24 h, the samples sown with fibroblasts were removed from the culture wells and fixed with 2% glutaraldehyde for 2 h. Then, the samples were dehydrated in a graduated series of ethanol washes and sputter coated with gold/palladium. Each sample was examined using a scanning electron microscope (JEOL JSM-6610LV SEM, Peabody, MA, USA) to elucidate cell-material interactions.

The expression of Col-1A and TGF-*β*1 was evaluated in fibroblasts treated with different cements, and GAPDH was used as housekeeping (Table 1) using endpoint PCR. The RNA was extracted by the trizol method, according to the manufacturer's specifications, and was quantified using a NanoDrop (Thermo Fisher, USA). The cDNA was synthesized using a high-capacity RNA-to-cDNA kit (Applied Biosystems) according to the manufacturer's instructions, and samples were kept at 4°C until use. The semiquantitative expression of Col-1A and TGF-*β*1 was determined using PCR Master Mix (Promega, Wisconsin, United States) in a thermocycler for 35 cycles with alignment temperatures of 58.1°C and 59.2°C, respectively. Once the PCR was completed, the product was loaded into a 2% agarose gel. Once the electrophoresis was completed, the gels were analyzed in a photoimager using the Bio-Rad Quantity One software.

The data of MTS cell proliferation and gene relative expression assays were submitted to normality (Shapiro-Wilk test) and variance equality (Levene test) assessments. The comparison procedures were carried out using Kruskal-Wallis test followed by the *post hoc* Mann-Whitney *U* test. Statistical significance was considered for values of *p* < 0.05 and were performed using SigmaPlot software version 11.0 (Systat Software Inc., San Jose, CA, USA).

## 3. Results

**Figure 1:**
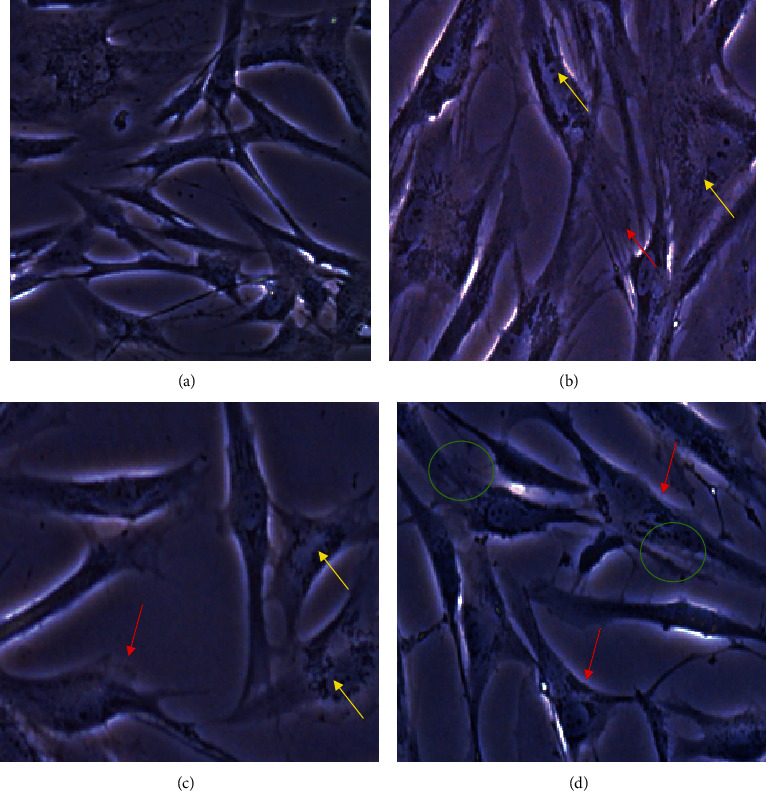
Micrographs of hFDP morphology at 24 h after application of the elutes of bioactive cements or fresh medium. Each cell culture was examined under an inverted optical microscope (Leica DM IL LED, Wetzlar, Germany) without any type of staining at a magnification of 40x. (a) Control group; (b) MTA-HP, shows alterations in cell membrane integrity (red arrow) an almost twofold increase in cell size and presence of intracytoplasmatic granules (yellow arrow); (c) Neo-MTA, increased presence of intracytoplasmatic granules, and the size of the cell is slightly increased (yellow arrow); (d) MTA-Ang, this treatment cell size is more conserved but decreases the amount of phyllopodia (red arrow) spindle cells with thin and branched cytoplasmic extensions were observed (green circle), alterations in membrane integrity and the presence of intracytoplasmatic granules.

The results shown in Figure 1 are representative of what was observed in most of the visual fields of the cell cultures treated with the different cements evaluated in this study.

The qualitative analysis of cytotoxicity is a first approach in trying to understand the beneficial or toxic effect that a chemical compound or material can cause when it comes into contact with cells, based on what is recommended by ISO10993-5-2009. Cell morphology in the control group (cells that did not receive any treatment) at 24 h is shown in Figure 1(a). Intracytoplasmic granules (arrow) and alterations in the cytoplasmic membrane (red arrows) were observed in cells treated with MTA-HP (Figure 1(b)). Among cells treated with Neo-MTA (Figure 1(c)), thin spindle cells were observed, and some cells contained intracytoplastic granules and showed increase in size (arrows). Among cells treated with MTA-Ang (Figure 1(d)), spindle cells with thin and branched cytoplasmic extensions were observed (circle), as were the presence of intracytoplasmic granules and cells with increases in size (arrows).

**Figure 2:**
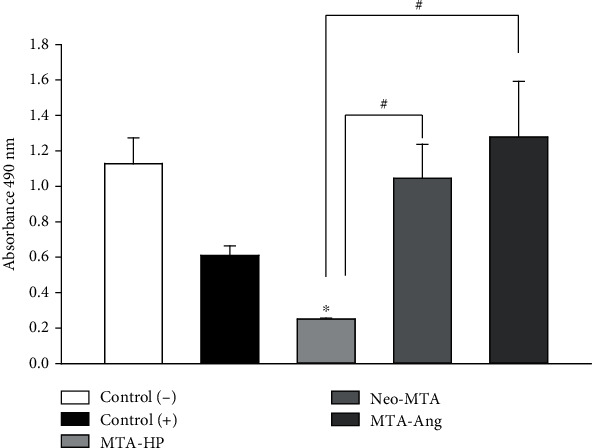
MTS assay on human dental pulp fibroblasts after contact with the different bioactive cements for 48 h. ∗ indicates a statistically significant difference compared with the control group; # indicates a statistically significant difference between the different groups (*p* <0.05).

The results obtained for cell proliferation are shown in Figure 2. At 48 h, the greatest cell proliferation was found in the MTA-Ang group, followed by the Neo-MTA group; there were no statistically significant differences in proliferation between these groups and the negative control group (cells that did not receive any treatment). Fibroblasts cultured in medium with MTA-HP showed significantly lower proliferation (*p* < 0.05) than those cultured in medium with MTA-Ang or Neo-MTA (decreased 82 and 17, respectively), while MTA-Ang had a positive effect on cell proliferation, increasing a little more than 10% in relation to negative control cells; the results obtained are validated by the formazan sales absorbance units obtained in the two controls, the negative one that represents the cells grown in optimal conditions and the positive control that represents the response of cells exposed to a cytotoxic agent.

**Figure 3:**
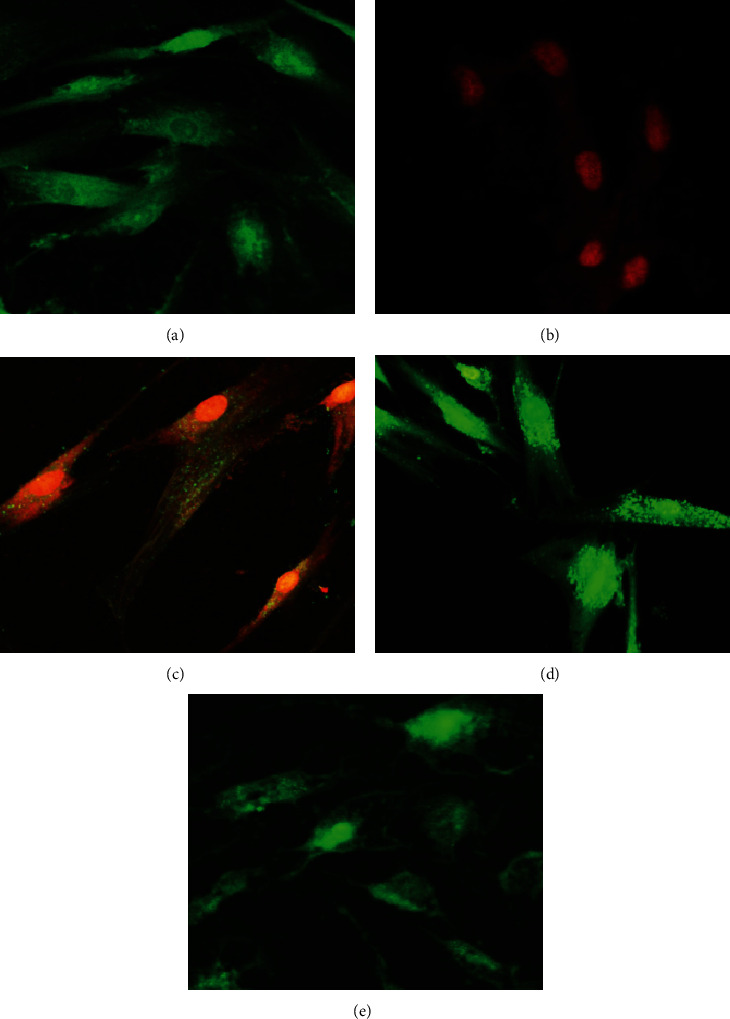
Fluorescence micrographs 40x of LIVE (calcein, green)/DEAD (EthD-1, red) assay on human dental pulp fibroblasts after contact with the different bioactive cements for 24 h. (a) Negative control cells in culture medium without any treatment. (b) Positive control cells treated with H_2_O_2_ and cells treated with the different bioactive cements. (c) MTA-HP, the cells are observed with a reddish color which indicates penetration of the ethidium homodimer characteristic of cell death. (d) Neo-MTA and (e) MTA-Ang, the cells are observed with an intense green coloration typical of cell viability.

The results for LIVE/DEAD assay are illustrated in Figure 3. At 24 h, intense green fluorescence produced by calcein was observed in the viable cells corresponding to the negative control group (cells that did not receive any treatment) (Figure 3(a)), while red fluorescence (indicating dead cells) was observed in the positive control group treated with hydrogen peroxide (cells treated with H_2_O_2_ to cause death); this red fluorescence was produced by EthD-1 inside the nuclei of cells with damaged membranes (Figure 3(b)). Fibroblasts cultured with MTA-HP were observed to have cell membranes permeable to EthD-1 and to be in the process of cell death (Figure 3(c)). Fibroblasts cultured with Neo-MTA were mostly viable cells (Figure 3(d)). Fibroblasts cultured with MTA-Ang were viable cells (Figure 3(e)). The results correspond with those obtained from the MTS experiment, where the MTA-HP sealant was the one that most decreased cell viability in the 48 h period.

**Figure 4:**
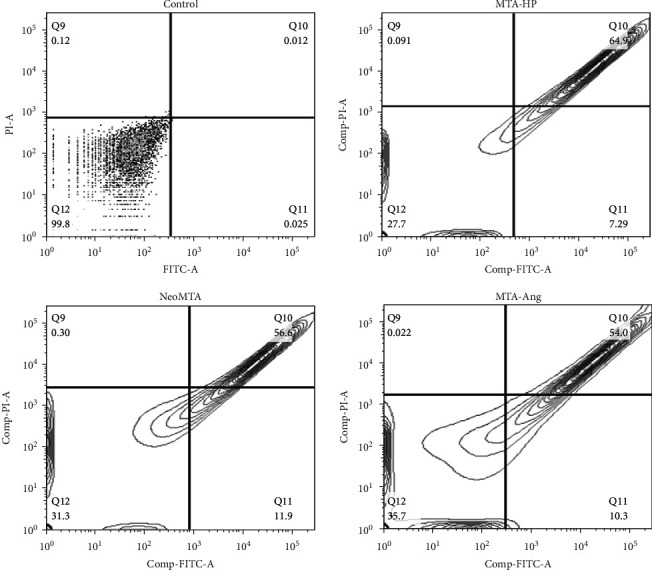
Apoptosis assay using flow cytometry after Annexin V-FITC/propidium iodide (PI) staining. Viable cells are in the lower left quadrant, early apoptotic cells are in the lower right quadrant, late apoptotic cells are in the upper right quadrant, and nonviable cells are in the upper left quadrant.

**Figure 5:**
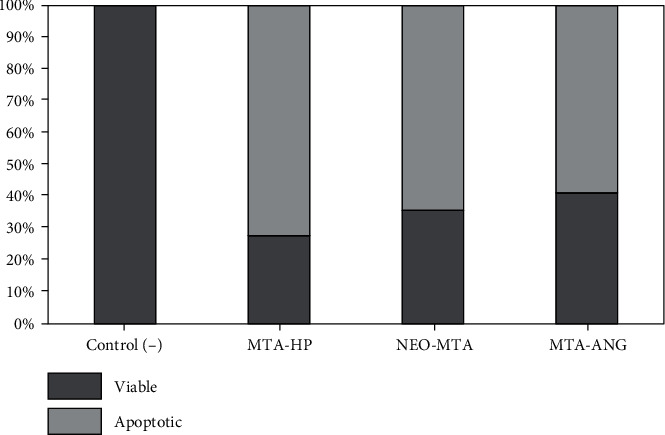
Effects of exposure of materials on the viability of dental pulp fibroblasts for 48 h assessed using flow cytometry. The cumulative diagram shows the percentage of viable, apoptotic cells.

AnnexinV binds specifically to the molecules of phosphatidylserine present in the plasma membrane of the cell; therefore, this assay can differentiate between cells that have intact membranes, and those with no intact membrane, which are classified as dead cells.  Figure 4 shows the apoptosis assay using flow cytometry after Annexin V-FITC/propidium iodide (PI) staining; viable cells are in the lower left quadrant, early apoptotic cells are in the lower right quadrant, late apoptotic cells are in the upper right quadrant, and nonviable cells are in the upper left quadrant. As shown in Figure 4, the exposure of cells to eluates from different materials resulted in significant reductions in the percentages of living cells in all groups compared to the negative control group after 48 h of treatment. As can be seen in Figure 4, a significant percentage of cells treated with MTA-HP is located in the upper right quadrant, which indicates, according to the manufacturers of the Annexin V-FITC assay, that cells present a certain degree of late apoptosis. The highest percentages of apoptotic cells were observed in the group of cells treated with MTA-HP eluate, followed by the group of cells treated with Neo-MTA eluate and finally the group of cells treated with MTA-Ang eluate (Figure 5). This experiment classifies more accurately the presence of apoptotic cells; therefore, it is an ideal complement for proliferation tests (MTS) and LIVE/DEAD assays.

**Figure 6:**
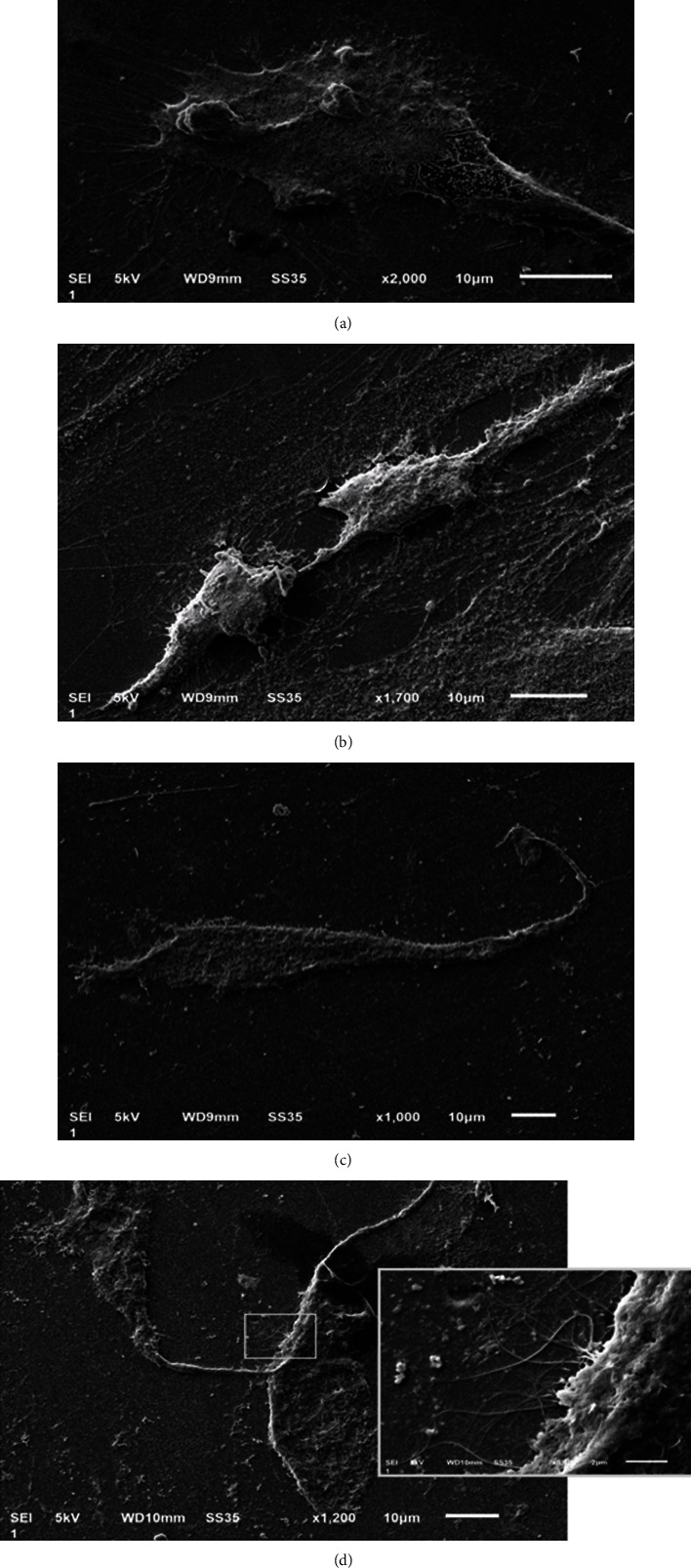
Ultrastructural analysis of fibroblasts with SEM. SEM micrographs of cultured fibroblasts in contact with the eluates of the bioactive cements for 24 h. 1000x. (a) Negative control group, fibroblast with a morphology and extensions in normal appearance. (b) MTA-HP group, cells are poorly dressed to the surface with a compact structure they were detached from the surface where they are growing. (c) Neo-MTA group, the cell has an adequate size; however, the shape and structure of the cell are altered as well as the number of focal contacts and phyllopodia that help the cell to adhere to the material on which they are growing are significantly reduced. (d) MTA-Ang group, the shape and structure of the cell are normal, robust focal contacts with a good generation of filopodias as seen in the magnification box. This micrograph highlights the focal contacts of the fibroblasts that represent a good adhesion to the surface that supports them; these characteristics can be extrapolated to a good response to the material to which it is exposed.

Figure 6 shows the cellular adhesion after the cells have been cultured on the eluates of the different cements analyzed after an incubation period of 24 h. In the negative control group (Figure 6(a)), superficial cell adhesion was observed, as well as the presence of filopodia and lamellipodia, which allow the appropriate cellular anchorage. When fibroblasts were cultivated with MTA-HP (Figure 6(b)), the cell changed its morphology, presenting less elongated and more rounded. Fibroblasts cultured with Neo-MTA (Figure 6(c)) showed a flattening of the central mass and a decrease in the phyllopodia and lamellipoidashing cells less stable than the control. Fibroblasts cultured with MTA-Ang (Figure 6(d)) were flattened and with longer filopodia spread and adhered to the surface.

**Figure 7:**
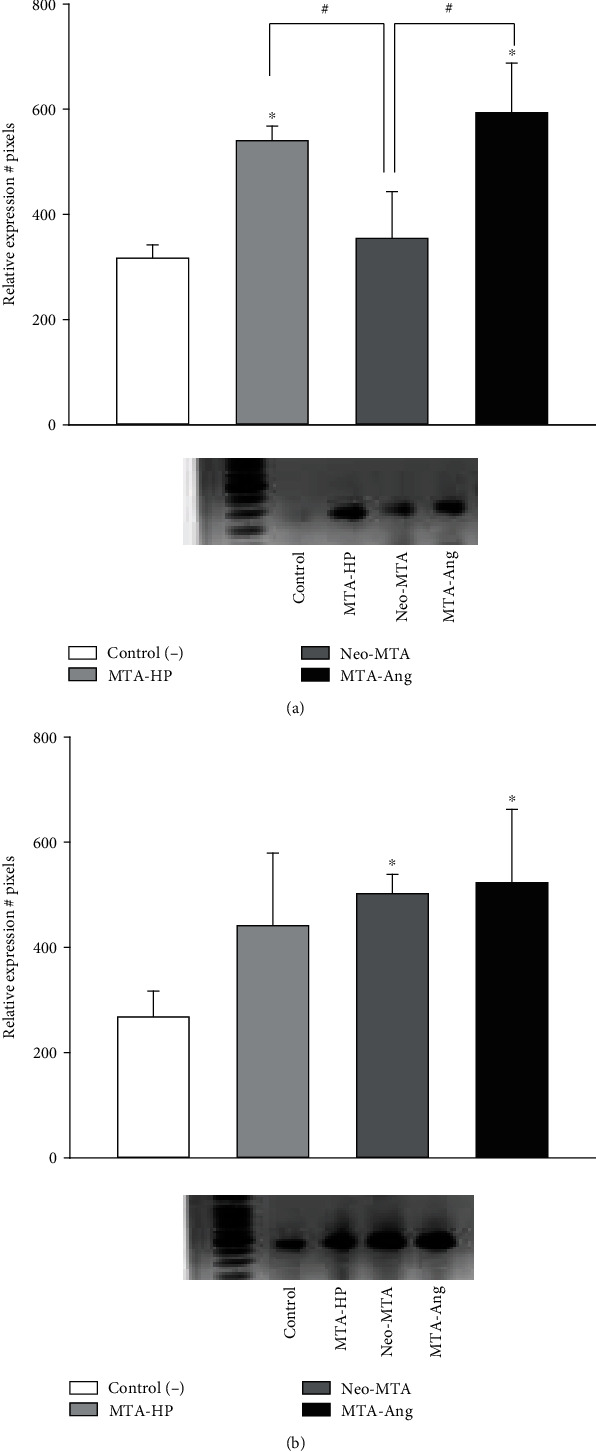
Results of the PCR endpoint assay to detect the expression of the (a) Col-1 and (b) TGF-*β*1 genes after 5 days of cell contact with the eluates of the different bioactive cements. ∗ indicates a statistically significant difference compared with the control group; # indicates a statistically significant difference between the different groups (*p* < 0.05).

The Col-1 gene was expressed at significantly higher levels in cell cultures treated with MTA-HP and MTA-Ang eluates than in the control group, with an increase in expression levels of 87% and 93%, respectively, or the cell group treated with Neo-MTA eluate with an increase in expression level of 22% (Figure 7(a)). With respect to the expression of TGF-*β*1, there were statistically significant differences in the groups treated with Neo-MTA and MTA-Ang eluates with an increase in expression levels of 75% y and 81%, respectively, compared to the control group (*p* < 0.05) (Figure 7(b)). MTA-HP has an increase in expression levels of 65%, but the differences were not statistically significant (*p* > 0.05).

## 4. Discussion

The objective of this study was to evaluate the biocompatibility and Col-1 and TGF-*β*1 expression modifier effects of MTA-HP and Neo-MTA cements in fibroblasts of human dental pulp and to compare these effects with those of MTA-Ang reference cement. These bioactive cements are proposed materials for vital pulp therapy that come into permanent contact with pulp tissue; therefore, they must exhibit low cytotoxicity and high biocompatibility, as well as the ability to induce the production of mineralized tissue [9, 10]. Among the principles that support vital pulp therapy are the cellular mechanisms involved in pulp repair, and the effect of MTA on cell viability requires study, since in clinical practice MTA could induce severe tissue damage and as a consequence treatment failure.

Cultures of human dental pulp fibroblasts, the main constituents of connective tissue and cells involved in the pulp healing process [11], were used to mimic clinical conditions. The use of primary human cells is relevant since differences between normal human tissue responses and murine nonspecific or immortalized tissue responses have been observed [12]. The toxic potential of the materials was evaluated after setting. Such analyses help elucidate the cytotoxic capacity of cement even after setting, and setting facilitates the microscopic visualization of treated cells [13].

The eluates from the materials used in this study were mainly used to evaluate whether the coating of the pulp with the materials evaluated would damage or irritate the pulp due to cytotoxic compounds that could pass through the blood clot and fluids physiological, inhibiting cell activity and growth [14] and causing tissue degeneration [15]. MTA has also been examined in new mixed formulations. Such formulations release large amounts of chemical by-products that can be toxic to cells in culture; however, in clinical settings, these by-products are likely to be diluted in interstitial tissue fluids and are eliminated through the vasculature, a biological response lacking in the cell culture model [16, 17]. One of the most important considerations affecting the cytotoxicity of MTA-based cements is the release of calcium hydroxide when the material is set; initially, the speed of the setting reaction is high, and the cytotoxic effects of the calcium hydroxide released in excess are observed [18]. As the material finishes setting, much of the alkalinity is lost, and cytotoxicity therefore decreases [19]. In the present study, an average setting time of 24 h was used so that the intermediate cytotoxicity could be evaluated between the time of greatest effect (freshly mixed MTA-based cement) and the time of fewest effects (completely set cement). Microscopic analysis of the effects of exposure of human dental pulp fibroblasts to the different types of MTA in the experimental environment used in this study revealed that the eluted materials used influenced cell morphology. These observations support the importance of conducting tests and determining the cytotoxic effects of different materials before use.

In the present study, the cytotoxicity of the different materials was evaluated by MTS proliferation assay. Cultured cells treated with Neo-MTA and MTA-Ang showed levels of cell proliferation similar to those of the control group and cell proliferation superior to that of cells treated with MTA-HP for 48 h. Neo-MTA is presented as a bioactive and nontoxic commercial material [20]. Compared to MTA-Ang, MTA-HP caused a decrease in cell proliferation; however, these results do not match those found in other studies showing similar biocompatibility between both types of MTA. Galarça et al. conducted a WST-1 trial on mouse L929 fibroblasts and found that MTA-HP- and MTA-Ang-treated cells showed similar cell viability after 24 and 48 h [21]. Cell culture studies with MTA have also shown that the cellular response to the material depends on many factors, such as the cell type, the chosen study duration, the use of a fresh or set material, the frequency of medium replacement, and the concentration of the material in the cell culture medium [22].

The cell membrane damage and the consequent cell death that the different materials could cause were evaluated by a LIVE/DEAD assay, and the results showed that at 24 h, MTA-HP caused damage to the cell membrane. The possible reason for this could be the leaching of certain toxic substances that adversely affect morphology of the cells [23]. On the other hand, Neo-MTA and MTA-Ang kept pulp fibroblasts viable. These results agree with the data obtained from the cell proliferation test.

Flow cytometry was also performed to analyze the mode of cell death (apoptosis). This test allows quantitative evaluation of apoptotic cells. FITC-bound Annexin V is a protein that, in the presence of calcium ions, binds specifically to the phosphatidylserine cell membrane, allowing the detection of apoptosis. The addition of PI to the mixture allows the simultaneous evaluation of cell membrane integrity. This dye does not pass through the lipid barrier, thus staining only cells with damaged cell membranes (apoptotic cells). PI penetrates dead cells, where it binds to nucleic acids and emits red and orange fluorescence when excited by blue light (*λ* = 420 nm). Apoptotic cells emit green fluorescence by exposing phosphatidylserine and Annexin on their surface. The intact (viable) cells are not stained. This test allowed us to distinguish three cell subpopulations: (i) necrotic cells, which were stained with PI and with Annexin V; (ii) viable cells, which lacked any staining; and (iii) apoptotic cells, which were stained to a greater or lesser extent with Annexin V. In this study, apoptosis of pulp fibroblasts was induced by the materials evaluated, mainly MTA-HP. These results were consistent with those of the MTS test. A previous study investigating the effect of the MTA dose on cell viability found that concentrations of MTA extracts greater than 2 mg/mL have toxic effects on cells [24]. The high percentage of necrotic cells can be attributed to the use of an eluate concentration of 2.5 mg/mL during the tests; these concentrations are based on the guidelines recommended by ISO 10993 [9].

Cell adhesion is a valid criterion for evaluating the biological effects of bioactive cements because binding to a substrate is the step that precedes proliferation, cell differentiation, and secretion of mineralized extracellular matrix. Regarding cell binding, previous studies have reported that observations of spindle-shaped cells in contact with biomaterials are good indicators of low material toxicity [25]. In the present study, the SEM assay revealed that cells treated with MTA-HP had less contact with the surface than those treated with Neo-MTA or MTA-Ang. Both morphology and adhesion are considered indicators of the biocompatibility of materials. In the present study, cell adhesion was increased among cells treated with Neo-MTA and MTA-Ang, and the results were consistent with the results of the cell proliferation assay. Some studies have reported that fibroblast-like MDPL-20 cells treated with extracts of MTA-based cement formulations adhere to glass substrates, displaying numerous long and slender cytoplasmic processes [26]. Future trials will be necessary to complete our findings.

The analysis of direct cytotoxics simulated the clinical situation in which mixed is directly applied to the surface of dental pulp. MTA acts as a “calcium hydroxide-releasing material,” but it does not cause as much caustic injury as a pure calcium hydroxide when in contact with tissue. Results of this study revealed that MTA-HP was cytotoxic in the short term. The cytotoxity activity could be related to excess calcium released. Barradas et al. reported that MTA-HP shows higher calcium release values at 24 and 72 h than MTA-Ang as well as higher solubility, which could be due to the plasticizer contained in the MTA-HP mixture liquid [27], which could underlie the cytotoxic effects of MTA-HP.

Calcium is one of the main constituents of most bone-promoting agents such that calcium release is associated with more bone formation in vivo. Calcium also plays an important role in cell signaling including cell survival and mineralization [28]. However, the increase in calcium concentration in a medium that contains cells can lead apoptosis by the intrinsic pathway, causing depolarization of the mitochondrial membrane, release of decytochrome C, and activation of the initiator caspases, and finally with it, the activation of the executing caspases leads the cell to death [29].

The use of bioactive materials to heal pulp tissue should increase the dentinogenic potential of pulp cells. In addition to biocompatibility, the influence of the materials on the main component of the pulp extracellular matrix, Col-1A, which has a direct role in the generation of hard tissue and the mineralization of dentin, was evaluated in this study. The transcription of Col-1A mRNA was upregulated in the cells treated with MTA-HP and MTA-Ang compared to the control and Neo-MTA-treated cells. In murine cell lines and cells of human dental pulp, materials containing calcium silicate decrease the mRNA expression of Col-1A [30, 31]; in this study, only Neo-MTA caused a decrease in expression.

TGF-*β*1 is of recognized importance in pulpal regeneration. Three isoforms of TGF-*β* (TGF-*β*1, TGF-*β*2, and TGF-*β*3) have been identified. In pulp fibroblasts, collagen matrix synthesis is induced by TGF-*β*1 and TGF-*β*2. TGF-*β*1 also plays a role in dental development and in the repair process by regulating proliferation, differentiation, and restorative dentinogenesis [30]. In the present study, the expression of TGF-*β*1 was higher in cells treated with Neo-MTA and MTA-Ang than in control cells. This finding can be correlated with the observed stimulation of fibroblast proliferation in the MTS trial. This finding is also consistent with the results of previous studies reporting the participation of TGF-*β*1 in the proliferation and recruitment of pulp fibroblasts [32].

It should be noted that the conditions reported in this study are a simulation of what is happening clinically. *In vitro* studies are limited by the fact that they do not take into account the interaction between cement components and interstitial tissue fluids. Furthermore, the amount of cement that comes into direct contact with the tissues varies from case to case, and therefore, it is unlikely that precise *in vitro* standardization can accurately simulate a common clinical situation. The clinically high success rate for MTA-based cement pulp capping or pulpotomy may be due to tissue homeostasis *in vivo* and is likewise able to reduce the initially high concentrations of calcium hydroxide and thus alkalinity [33]. Our results showed the effect of materials for clinical use on the limitations of cell line culture; for these reasons, more *in vivo* research is needed. It is required, in the future, that studies evaluate quantitatively the focal contacts that cells developed when treated with different materials.

## 5. Conclusions

The present study demonstrated that Neo-MTA and MTA-Ang presented similar biocompatibility, and both showed greater biocompatibility than MTA-HP. MTA-HP and MTA-Ang increased Col-1A gene expression, and Neo-MTA and MTA-Ang increased TGF-*β*1 gene expression in a similar way.

## Figures and Tables

**Table 1:** Sequences, Tm, and sizes of primers used for gene amplification.

Gen	Tm	Sequences (5′-3′)	Fragment size
GAPDH Fw	65.2°C	CCATCAATGACCCCTTCATTGACC	435
GAPDH Rv		TGGTCATGAGTCCTTCCACGAT	
Col-1A Fw	58.1°C	GATTCCCTGGACCTAAAGGTGC	230
Col-1A Rv		AGCCTCTCCATCTTTGCCAGCA	
TGF-*β*1 Fw	59.2°C	ACATGGAGCTGGTGAAACGGAA	484
TGF-*β*1 Rv		AAAGACAGCCACTCAGGCACTCAGGCGTAT	

## Data Availability

All data used to support the findings of this study are included within the article.
